# The Treatment of Genito-Urinary Diseases in New York City

**Published:** 1892-09

**Authors:** J. Arthur Chiles

**Affiliations:** Atlanta, Ga.


### *CORRESPONDENCE.*

---

ATLANTA, GA., August 20, 1892.

### THE TREATMENT OF GENITO-URINARY DISEASES IN NEW YORK CITY.

During the summer, spent in the Hospitals of New York City, studying genito-urinary diseases, I had an opportunity of observing carefully the advances which have been made in that branch of medical science, and to watch closely the favorite modes of diagnosis and treatment of our professional brethren of that great medical center, who have spent many years in the study of diseases of this class; and I hope to be able to give a few points which will be of interest and profit to the readers of your journal.

As is well known, slight stricture of the urethra is the most frequent cause of obstinate and persistent gleet, and it is in these cases where our injections fail to give the hoped-for results, and the use of the steel sound, though successful in many cases, does not always effect a cure. These are the cases which call for an operation which, though simple in itself, and in which the mortality is very small, may suddenly develop grave symptoms, that human skill cannot subdue, and in a few hours may prove fatal. I speak of the operation of internal urethrotomy, for anterior stricture of large calibre; and the care with which the New York surgeons of wide experience prepare their patients for this simple operation shows with what dread they look upon urethral fever; and they claim that by the precautions and proper preparation of patient they reduce the danger to the minimum, and rarely have the development of this dread complication. For three days before the operation, no matter what the general health of patient may be, he is confined to bed, put on a restricted diet, and given 10 gr. boracic acid three or four times a day, and if there is much discharge from urethra or inflammation of the bladder these are irrigated two or three times a day with saturated solution of boracic acid, and the night before the operation he is given a brisk cathartic (usually saline). Just before the operation the urethra is thoroughly irrigated, and the patient is ready to undergo the operation with the smallest degree of danger. After the operation, if there is reason to suspect that the patient has been exposed to malaria he is put upon quinine in large doses; otherwise unless there is pain or other symptoms calling for treatment the patient is simply put to bed and kept as quiet as possible for from three to six days, when the danger is usually passed. I asked Dr. Bangs, a man of wide experience, how he treated urethral fever, and his answer was, "I treat it before the operation."

But there is another class of cases in which we have persistent gleet, and in which we can find no organic stricture, or perhaps no stricture at all, but in many of these cases there is spasmodic stricture, which will disappear when the cause, viz., granulations growing upon the urethral wall, has been removed. In some of these cases the diagnosis is very difficult, or even impossible, without the proper instruments for inspecting the urethral canal. For this purpose most surgeons use Leiter's electric urethroscope, for the reason that they have had nothing better. Leiter's instrument is large and heavy, and, an objection which is more serious, because as a rule we have more strength than money, is very expensive. But there is now to be had, at a small expense, a new form of electric urethroscope, which has been ingeniously contrived by Dr. William K. Otis, of New York. It is light, but *one-sixth* the weight of Leiter's, very simple of construction, and gives more room for the use of other instruments. With this little instrument we can examine and view the walls of the urethra with as much ease as we can the nasal cavities, and with a slender silver probe, upon which a small piece of absorbent cotton is twisted, can make applications of silver nitrate, etc., directly to the granular surface, and it is claimed by those who have tested this mode of treatment that they rarely fail to get favorable results. Another mode of treatment in these cases which is much used in the hospitals of New York, and often gives good results, is to apply directly to the granular surface with Ultzmann's deep urethral syringe two or three drops nitrate silver solution, of the strength of from 5 gr. to 1 oz. to 48 gr. to 1 oz., making the application about every third day.

There seems to be a tendency to lay aside the nauseous internal remedies formally used in the treatment of acute gonorrhœa and to rely now upon local treatment, especially irrigation with hot water, to which some mild antiseptic is added, with the internal administration of remedies which tend to disinfect the urinary tract, but do not upset the stomach. In cases of simple urethritis, in which, as a rule the gonococcus is not found, irrigation twice daily will almost always stop the discharge inside of ten days. At the Post-Graduate Hospital we had two cases of urethritis which interested me greatly. One was in a child about three years old, the other a boy of eight or nine, in neither of which could we trace source of infection, if it was infectious, nor could we after careful examination discover the presence of the gonococcus. Both responded promptly to irrigation.

In syphilitic and chancroidal cases our instructors were exceedingly careful not to make a mistake in diagnosis, for they seemed to realize, as every physician who treats such cases should, the seriousness of such an error. Save in the most typical cases of chancre, and even in many of these, they wait for secondary symptoms before putting the patient on constitutional treatment.

The treatment in these cases seemed to me to be almost routine, and was very much in line with Dr. Keyes' "Tonic Treatment of Syphilis." Of course, one cannot always judge from hospital cases how a man will treat cases in private practice, but in nearly all the cases seen the treatment for the early stage was : Hydrarg. protiodide, ¼ gr. ; ferri. reduct. gr. i ; m. ft. pil. no. 1. Sig. One pill t. i. d. For the late stage potas. iod., of course. For mucous sours in mouth and throat, local applications silver nitrate gr. xx to gr. xl, to an ounce of water. Who knows of a better treatment if the patient is carefully watched and modification made as required ?

The cystoscope of Leiter is coming more into use, and is often of great aid in the diagnosis of diseases of the bladder, especially in cases of hæmaturia, where we find it difficult or impossible otherwise to ascertain the source of the hemorrhage.

Dr. Bangs had a case which he had studied carefully for weeks, but was unable, with the ordinary means of diagnosis, to tell where the blood came from, but after thoroughly washing out the bladder and filling it with clear water, the cystoscope was passed in and the blood was seen escaping from one of the ureters. I give this case simply to illustrate the utility of the instrument.

I believe the instrument is not made in this country, and like all foreign goods upon which we pay duty, is expensive and is rather easy to get out of order.

Cutting for stone in the bladder is not so frequently resorted to as in the days of Gross and others who have given the subject such careful study. The favorite operation in favorable cases is "lithotrity at one sitting," after the manner of its inventor, Dr. Henry J. Bigelow, of Boston. Of course, in cases of stricture of urethra, greatly enlarged prostate, high degree of inflammation of bladder, very large stone or stone incysted or too hard to be crushed, the cutting operation is resorted to, and then we must decide between the perineal and the suprapubic operations, the latter of which is growing in favor, but will probably never supplant entirely the perineal operation, especially in children, and for stones of small size in the adult.

J. ARTHUR CHILDS, M. D.

---

### ACUTE TONSILLITIS.

Dr. A. Martin (*La Semaine medicale*, No. 55, 1891) speaks highly of the following:

℞. Acid carbolic,
Camphor, aa gram 1.
Glycerine,
Aq. destillat., aa grams 50.

Three or four local applications a day.

